# Cumulative Ecological Impact of Cascade Hydropower Development on Fish Community Structure in the Main Stream of the Xijiang River, China

**DOI:** 10.3390/ani15040495

**Published:** 2025-02-10

**Authors:** Yuansheng Zhu, Jiayang He, Fangyuan Xiong, Zhiqiang Wu, Jiajun Zhang, Yusen Li, Yong Lin, Anyou He, Dapeng Wang, Yaoquan Han

**Affiliations:** 1 Pearl River Water Resources Protection Scientific Research Institute, Guangzhou 510611, China; zhuyuansheng2024@163.com (Y.Z.); xiongfy2023@163.com (F.X.); 15823210153@163.com (J.Z.); 2 Institute of Hydrobiology, Chinese Academy of Sciences, Wuhan 430072, China; hejiayang@ihb.ac.cn; 3 College of Environmental Science and Engineering, Guilin University of Technology, Guilin 541004, China; 4 Guangxi Institute of Fisheries, Nanning 530021, China; listen2019f@163.com (Y.L.); linnn2005@126.com (Y.L.); heanyou2000@aliyun.com (A.H.); oucwdp@163.com (D.W.); hyqao@sina.com (Y.H.)

**Keywords:** fish-based index of biotic integrity, environmental stress evaluation, river health, fish resource conservation

## Abstract

In recent decades, dams worldwide are increasingly constructed in a row along a single river or basin, thus forming reservoir cascades, and in turn producing cumulative ecological effects along these areas. The use of multimetric indices (MMI) based on fish assemblages to assess the ecological health status of rivers and lakes has also been extensively developed. However, to date, there are no studies that employ MMI for the identification of the cumulative effects of reservoir cascades. The aim of this study was to develop a new Fish-based Index of Biotic Integrity (F-IBI) that can effectively identify the cumulative effects of reservoir cascades on fish assemblages in two important habitats (the free-flowing reach between reservoirs and the transition zone in the reservoir). Fish assemblages from 12 sites were sampled along the cascade reservoirs in the Xijiang River, China. First, through screening for redundancy, precision, and responsiveness of the candidate metrics, a new F-IBI based on ecological trait information of fish species composition was developed to estimate the ecological status of all sites. F-IBI scores exhibited an obviously downward trend from upstream to downstream in a reservoir cascade, and the transition zones in the reservoir displayed significantly lower F-IBI scores than the free-flowing reaches between reservoirs. Secondly, using Random Forest models, it was shown that the F-IBI can effectively identify the cumulative effects of the reservoir cascades on fish assemblages. Furthermore, we also demonstrated metric-specific responses to different stressors, particularly the multiple reservoir cascades, which showed the following: (1) The F-IBI index system developed based on the Random Forest model can effectively identify the superimposed effects of cascade power stations on fish integrity changes, with the cumulative time effect and the GDP index of river segments playing a key role; (2) To effectively protect the fish resources in the main stream of the Xijiang River, where priority should be given to the habitat of the natural flowing river sections between the reservoirs. At the same time, environmental regulatory policies should be formulated accordingly based on the human development status of each river section.

## Simple Summary

The construction of multiple dams along a single river or within a basin has become increasingly common worldwide in recent years, resulting in the formation of reservoir cascades and cumulative ecological impacts. This study developed a Fish-based Index of Biotic Integrity (F-IBI) to assess the cumulative effects of reservoir cascades on fish communities. Fish samples were collected from 12 sites along the Xijiang River basin in China. The findings revealed a significant decline in F-IBI scores from upstream to downstream, with transition zones within reservoirs exhibiting notably lower scores compared to the free-flowing river sections between reservoirs. Using Random Forest models, the F-IBI effectively identified the combined impacts of reservoir cascades on fish assemblages, highlighting the importance of cumulative time effects and the GDP index of river segments. This study recommends prioritizing the protection of natural river habitats between reservoirs and formulating environmental regulations tailored to the level of human development in each river segment to effectively conserve fish resources in the Xijiang River basin.

## 1. Introduction

The perpetuation of natural hydrological regimes has been instrumental in sculpting an evolutionary landscape of aquatic fluidity. This process facilitates the establishment of structural habitat units, the augmentation of ecological connective pathways, and the fostering of interspecies interactions. These mechanisms have led to the emergence of species adept at thriving in diverse aquatic environments, culminating in the genesis of complex and diverse fluvial biotic assemblages and ecosystem architectures [1,2]. Moreover, the conservation of intrinsic hydrological attributes across both temporal and spatial spectra is imperative for safeguarding the operational integrity of ecosystem services specific to aquatic environments and for maintaining the holistic integrity of aquatic biota [3]. Crucially, the preservation of the longitudinal hydrological continuum (from headwaters to the river mouth) and lateral hydrological connectivity (between riverine systems and adjacent floodplains) is of paramount importance for the completion of life cycles among a host of aquatic taxa, including anadromous and catadromous fish species [4,5]. Recent studies in major river basins such as the Mississippi, Mekong, and Yangtze have highlighted the global ecological impacts of gradient hydropower stations, particularly through the formation of cascading reservoir systems. These developments have raised concerns about their cumulative effects on fish community structures and ecosystem health over time [6,7].

The implementation of anthropogenic structures such as cascading artificial dams has significantly perturbed pristine riverine habitats. These structures modulate fluvial flow regimes, transforming native hydrodynamic and thermodynamic characteristics, and profoundly impacting biodiversity, particularly the structural and functional dynamics of ichthyofaunal communities [8,9]. As a result, ichthyic species and other aquatic life forms—encompassing planktonic organisms, hydrophytes, and benthic entities—serve as sensitive bioindicators that reflect anthropogenic pressures, including riverine impoundments and declines in aquatic trophic quality [10,11]. The Fish-based Index of Biotic Integrity (F-IBI), proposed by Karr in 1981, stands out as a comprehensive methodology for assessing these impacts. It synthesizes data spanning from individual organisms to populations, communities, biogeographical distributions, and ecosystemic levels into a single evaluative metric [11]. Over the years, advancements in F-IBI methodologies have enabled its application across diverse regions, providing a more holistic biological perspective in reflecting the health status of riverine systems compared to traditional physicochemical parameters [10,12]. However, challenges remain in addressing the specific impacts of cascading reservoir complexes on fish community structures within individual river segments [13,14].

To address these challenges, the global scientific community has adopted bioassessment metrics predicated on the structural and functional traits of aquatic biotic communities to evaluate the extent of anthropogenic perturbations upon aquatic ecosystems [12,13]. Notably, fishes, with their extended longevity, enhanced migratory faculties, expansive habitat range, and elevated trophic positions, are particularly robust bioindicators for monitoring and assessing anthropogenic disturbances across broader ecological timescales and spatial dimensions [14]. By employing the F-IBI framework, researchers have effectively analyzed anthropogenic impacts on aquatic ecosystems and their structural and functional ramifications, providing a potent scientific foundation for informed management decisions [15,16]. However, limited research exists on the specific role of hydrological environmental filtering versus anthropogenic pressures in influencing biotic integrity within inter-reservoir lotic segments and tailwater transition zones.

Within cascading reservoir systems, four distinct habitat typologies have been identified: lotic river segments (bridging upstream dams with downstream reservoirs), lentic river habitats, transitional zones, and lacustrine habitats within reservoirs [17]. Despite some progress, factors such as the spatial distribution of dams and the proximity of sampling sites to dam and lacustrine habitats remain underexplored in terms of their influence on biotic integrity. Even with the preservation of lengthy natural lotic habitats between reservoirs, maintaining high biotic integrity scores remains challenging, raising questions about whether the reduced scores stem from anthropogenic pressures or hydrological environmental filtering [16].

Thus, this research is predicated on the following hypotheses: (1) River segments with perennial flow between cascade stations are more conducive to ichthyological biotic integrity than the reservoir tailwater transition zones; (2) Anthropogenic pressures resulting from cascade hydropower development are more influential than hydrological environmental filtering in affecting the biotic integrity of both the inter-reservoir lotic segments and the tailwater transition zones; (3) The F-IBI framework is capable of effectively discerning the cumulative ecological effects of human pressures on fish community structures. To test these hypotheses, this study developed a novel F-IBI framework to identify the predominant pressures—whether anthropogenic or environmental filtering—that govern the cumulative impacts on fish community structures. This framework provides a robust scientific foundation for informed management and conservation strategies in river systems impacted by gradient hydropower development.

## 2. Materials and Methods

### 2.1. Sampling Methods and Locations

**Figure 1:**
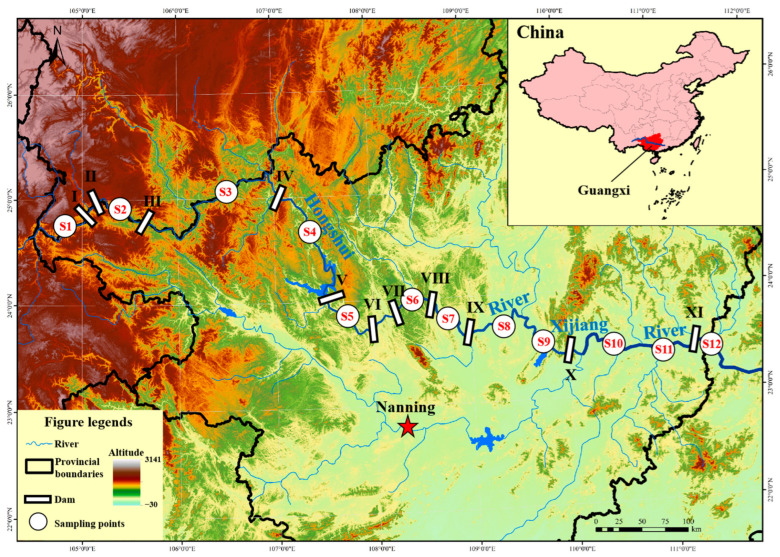
Distribution map of 12 sampling points in the mainstream of the Xijiang River. Note: 11 constructed hydropower stations, arranged downstream as follows: Tianshengqiao First Grade (**I**), Tianshengqiao Second Grade (**II**), Pingban (**III**), Longtan (**IV**), Yantan (**V**), Dahua (**VI**), Bailongtan (**VII**), Letan (**VIII**), Qiaogong (**IX**), Datengxia (**X**), and Changzhou (**XI**).

In consideration of the ichthyofaunal composition, the habitat characteristics of the Xijiang River mainstream, and a comprehensive evaluation of the locations and reservoir lengths of various hydropower stations along the main river, we strategically established 12 sampling sites (Figure 1). Fish samples were collected at each site during four distinct seasons: July–August 2022 (summer), November 2022 (autumn), February 2023 (winter), and May 2023 (spring).

Samples were obtained through fishing vessel operations and supplementary market purchases, employing 97 hired fishing vessels. Different fishing methods were applied based on the diverse habitat characteristics of each site: in shallow and fast-flowing waters, single-layer gillnets (20–50 m in length, 1–2 m in height, with mesh sizes of 1–6 cm) were used; in deeper and slower waters, fishing was primarily conducted using fyke nets (0.3 m wide, 0.25 m high, 8 m long, with a mesh size of 4 mm) supplemented by gillnets. To ensure data comparability, the specifications of the nets, deployment methods, and duration of each sampling effort were standardized. Each cross-section at a sampling site was divided into three water layers according to varying water depths for sample collection, ensuring a consistent sampling duration of approximately 12 h.

Fish samples purchased from fisheries workers were accompanied by detailed records of the fishing trip, including time, location, and equipment used, allowing for a comprehensive and complete reflection of the fish community structure. Following sample collection, immediate on-site identification of species was conducted based on the ‘*Freshwater Fish of Guangxi*’ (Second Edition) [18]. The total length was measured using a digital caliper with an accuracy of 0.1 cm, and the weight was measured using a portable electronic scale with a precision of 0.1 g. Samples that could not be easily identified on site were measured and photographed before being preserved in formalin for further analysis and identification in the laboratory.

### 2.2. Study Area

The main stream of the Xijiang River, sequentially named the Nanpan River, Hongshui River, Qian River, Xun River, and Xi River from upstream to downstream, collectively referred to as the Xi River, primarily traverses the Guangxi Zhuang Autonomous Region. Reservoirs can typically be divided into three different habitat types based on habitat differences: the riverine zone, the transition zone, and the lacustrine zone [19]. The riverine zone is the lotic habitat of the reservoir reach; the lacustrine zone is the area closest upstream to the dam site within the reservoir, characterized by an absence of noticeable flow velocity; the transition zone lies between the riverine and lacustrine zones, with features of low or negligible flow velocity during periods of high water level operation and fast flow velocity with the highest nitrogen fixation efficiency during low water level operation [20,21]. The study area encompasses 11 hydropower stations (Figure 1). Sampling sites were strategically located upstream and downstream of each dam. Based on variations in the water surface area associated with each hydropower station and the habitat characteristics of the sampling sites, these locations were classified into Natural Free-flowing Reach of Dams (NRAD) and Transitional Region in Reservoir (TR). For detailed information on the changes in water surface area associated with each station, please refer to Supplementary Material S1. Six sampling sites, including S3 and S8 to S12, are situated within the lotic river segments between the dam and the reservoir, while the other six sites, including S1, S2, and S4 to S7, are located in the transition zones of different power stations (Table 1).

### 2.3. Fish Data Acquisition

Fish species composition data for the 12 sampling sites around the 1980s (prior to the cascade hydropower development on the main stream of the Xijiang River) were collected from the ‘Report on the Survey and Research of Inland Water Fishery Natural Resources in Guangxi Zhuang Autonomous Region’ [22], ‘The Freshwater Fishes of Guangxi Zhuang Autonomous Region’ [23], and ‘The Cyprinidae of China’ [24]. All fish Latin names were cross-checked with the FishBase database. Ecological traits such as endemism, flow preference, diet, and spawning characteristics were determined based on published papers, monographs, and databases. Twenty-five ecological trait characteristics were selected for ecological trait categorization, including endemic fish, pelagic fish, midwater fish, benthic fish, superior mouth fish, terminal mouth fish, inferior mouth fish, carnivorous fish, omnivorous fish, herbivorous fish, planktivorous fish, benthivorous fish, migratory fish, still water fish, eurytopic fish, rheophilic fish, flat-bodied fish, tubular fish, laterally compressed fish, spindle-shaped fish, benthic egg fish, adhesive egg fish, pelagic egg fish, drifting egg fish, and fish with special spawning methods (Supplementary Material S2). These ecological trait categories were set as 25 parameters, each containing binary presence/absence information (0/1, where 0 indicates the species does not possess the ecological trait characteristic, and 1 indicates the species does possess the specific ecological trait characteristic). The current fish data were classified and organized based on the results of this survey, as detailed in Supplementary Material S3.

### 2.4. Data Statistics and Analysis

#### 2.4.1. Fish Data

Historical Fish Data: Firstly, the 0/1 matrix data of historical fish distribution were standardized using a z-score transformation before the clustering tendency was quantitatively assessed using Hopkins statistics [25]. The assessment procedure is as follows: 1. Randomly select n samples from N total samples and for each sample, find the nearest vector in the sample space, calculating the distance between them, represented by the set *r* = [*r*_1_,…, *r*_n_]; 2. Repeat step 1 once, with the distance set represented by *r*’ = [$r_{1}^{\prime }$,…,$r_{n}^{\prime }$]; 3. Calculate the Hopkins statistic using the following Formula (1):

(1) $$H=\frac{\sum_{i=1}^{n}r_{i}^{\prime }}{\sum_{i=1}^{n}r_{i}+\sum_{i=1}^{n}r_{i}^{\prime }}$$

If *H* is around 0.5, then the sample space tends to be uniformly distributed, i.e., there is no clustering tendency (clusters are not distinct), and there is no value in clustering; if *H* tends towards 1 or 0, it indicates that there is a significant clustering tendency in the sample space, and there is value in clustering.

Secondly, hierarchical cluster analysis (CA) was employed to explore the spatial distribution patterns of fish compositions. The Jaccard index was used to calculate the similarity between different sampling sites, forming a similarity matrix. Subsequently, the clusgap algorithm was used to determine the estimated number of optimal clusters [26]. On this basis, the fish species distribution data from different sampling sites belonging to the same cluster were integrated to form updated fish composition information corresponding to that cluster. Using the updated fish composition information, the total number of fish species for each cluster can be calculated, representing the expected total number of species for the various sampling sites. In other words, different aquatic regions within the same cluster will share a common expected total number of species. All the above analyses were conducted using the ‘factoextra’ and ‘cluster’ packages in R for analysis and visualization.

#### 2.4.2. Selection of Sensitive Indicators

Redundancy Analysis: Spearman’s correlation was applied to 25 ecological habit traits to identify significant collinearity (r ≥ 0.60). Traits with lower correlations to endemic fish species in the Pearl River were retained as initial candidate indicators for constructing a biotic integrity index [11,16].

Discrimination Power Screening: Initial candidate indicators were screened for their ability to discriminate human disturbances. Reference values were based on historical fish species data (pre-1980s), while impaired values were derived from current surveys (2006–2023). Two hypotheses were proposed: (1) significant fish community structure changes post-cascade hydropower development, and (2) impaired biotic integrity post-development. Indicators were scored (IQ = 0 to 3) based on interquartile range overlap between reference and impaired values, with IQ = 3 indicating very strong discrimination power [16].

Environmental Stress Response Analysis: Retained indicators were assessed for consistency with temporal trends. Indicators with post-development values uniformly lower or higher than pre-development values were considered sensitive to environmental stress; others were removed [11,16].

#### 2.4.3. River Section F-IBI Scores and Scoring Grade Classification

The reference values for the selected sensitive indicators were re-derived based on the results of cluster analysis of the fish species composition data; their current values were obtained from the status quo survey data of each sampling river section after the development of the cascade hydropower stations. The score for each sensitive indicator is obtained using a ratio method. If the value of the sensitive indicator decreases with stronger disturbance, the F-IBI score for that sensitive indicator at each sampling river section (SE1) is calculated using the following Formula (2) [16]:

SE1 = C/Rgroup(2)

where C is the current value of the sensitive indicator at each sampling river section, and Rgroup is the reference value of the sensitive indicator corresponding to the cluster group of the sampling river section.

If the value of the sensitive indicator increases with stronger disturbance, the F-IBI score (SE2) is calculated using the following Formula (3):

SE2 = (Max − C)/(Max − Rgroup)(3)

By summing the F-IBI scores of all sensitive indicators, the total F-IBI score for each sampling river section is obtained. The expected total F-IBI score for each sampling point is derived by multiplying the number of sensitive indicators by 1. Finally, based on the 95th percentile of the expected total score, the F-IBI score grades are divided into five categories: scores greater than the 95th percentile are classified as ‘Excellent.’ The scores between 0 and the 95th percentile are then evenly divided into four parts, resulting in the grades ‘Good’, ‘Fair’, ‘Poor’, and ‘Very Poor’ [27].

#### 2.4.4. Comparative Analysis of F-IBI Score Differences

Initially, the Kolmogorov–Smirnov test was employed to assess the normality and homogeneity of variances [28]. Subsequently, an independent samples *t*-test was conducted to examine the differences in the proportion of fish species numbers of different ecological types, as well as the F-IBI (Fish Index of Biotic Integrity) scores between two habitat types (NRAD and TR).

#### 2.4.5. Relationship Between F-IBI Scores and Environmental Factors

To evaluate the impact of environmental stress (human pressure and environmental filtering) on fish community structure before and after the development of cascade hydropower stations, a Random Forest (RF) model was employed. The RF model, based on classification tree algorithms, is widely used in machine learning for analyzing relationships between biological and abiotic factors, unaffected by multicollinearity and robust for missing or unbalanced data [16,29].

The model used Fish-based Index of Biotic Integrity (F-IBI) scores as the dependent variable and 11 abiotic variables as explanatory factors. Human pressure variables included Gross Domestic Product (GDP), Land Use Ratio (LUR), and Cumulative Time Effect of Power Stations (CTP). Environmental filtering variables comprised Water Area (WA), Annual Average Runoff Volume (VR), Annual Air Temperature (TEM), Annual Average Precipitation (PRE), Natural Free-Flowing River Segments between Adjacent Dams (NRAD), Habitat Type (HT), Drainage Network Density (DWN), and Elevation (ALT). Data were derived from Landsat TM imagery, ArcGIS 10.2, Bigmap 2024v3.1.10716, and the NASA POWER dataset. Air temperature substituted for water temperature due to their high correlation.

Air temperature data, taken at two meters above the ground (temperature at 2 m), and precipitation data are acquired through the nasapower R package [30] using the function get_power from the National Aeronautics and Space Administration (NASA). Multi-year average runoff volume data (1961–2018) are obtained through Panoply software version 5.2.9 from the China Natural Runoff Dataset version 1.0 (1961–2018) [31]. The 1 km grid GDP data for each river section is sourced from the National Tibetan Plateau Data Center (http://data.tpdc.ac.cn, accessed on 6 July 2023) in the Historical GDP Spatial Distribution Kilometer Grid Dataset of China (1990–2019) (https://doi.org/10.12078/2017121102). HT represents two different types of habitats: NRAD and TR (Table 1), as an ordinal categorical variable (NRAD, 1; TR, 2). CPT is an ordinal categorical variable reflecting the cumulative effect of connectivity over time after the development of cascade hydropower stations, mainly characterizing the cumulative effect of connectivity over time after the construction of cascade power stations. The formula for the cumulative effect of connectivity over time for the i-th river section (TRCIi) is based on the River Connectivity Index (RCI) [32] and optimized with a time-weighted adjustment (4):

(4) $${\mathrm{TRCI}}_{i}=\frac{\sum_{k=1}^{t}100\times \sum_{j=1}^{n}\frac{2^{r_{i}}l_{i}}{L}\times \frac{2^{r_{j}}l_{j}}{L}p_{ij}}{t}$$

(5) $$L=\sum_{i=1}^{n}2^{r_{i}}l_{i}$$

(6) $$p_{ij=\prod_{m=1}^{M}p_{m}}$$

In the formula, *t* represents the duration of water storage (in years) for the first cascade hydropower station to store water; *k* is the first year of water storage; n is the total number of river segments; *r*_i_ is the river rank of segment *i* (all are first-order rivers); *l*_i_ is the length of segment *i*, in kilometers; *L* is the total length of river segments after weighting by river rank (5); *M* is the number of barriers between segment *i* and *j*; pm is the passability of the m-th barrier (complete barriers are assigned a value of 0.00; those with fish passage facilities are assigned a value of 0.25), with a range from 0 to 1; *p_ij_* is the passability between segment *i* and *j* (6). The above analyses were conducted using the Kolmogorov–Smirnov test to assess the normality and homogeneity of variances.

Variables were log10 (x + 1) transformed, and the leave-one-out method was used for model validation. Importance metrics (%IncMSE and IncNodePurity) identified the top four environmental variables influencing F-IBI scores, which were fitted using linear or polynomial regression. Statistical analyses were conducted in R, with significance at *p* < 0.05. The RF model provided insights into the drivers of fish community changes, demonstrating its effectiveness in ecological assessments [16,33].

## 3. Results

### 3.1. The Spatial Distribution Characteristics of the Historical Fish Species Composition at Each Sampling Site

A total of 15,674 fish were collected, belonging to 14 orders, 34 families, 106 genera, and 139 species, of which 120 were indigenous species and 19 were exotic species. At the order level, Cypriniformes were predominant with 79 species, accounting for 56.83% of the total species, followed by Perciformes with 28 species, accounting for 20.14%. At the family level, Cyprinidae was dominant with 65 species, accounting for 46.76%, followed by Cobitidae with 9 species, accounting for 6.47%. The dominant species throughout the year were *Coptodon zillii*, *Squaliobarbus curriculus*, *Cyprinus carpio*, and *Cirrhinus molitorella*; in the upstream river sections, the dominant species were *C. carpio*, *C. zillii*, and *Oreochromis niloticus*; in the midstream sections, *Ptychidio jordani*, *Discogobio tetrabarbatus*, and *Acrossocheilus longipinnis* were dominant; and in the downstream sections, *Megalobrama terminalis*, *Parabramis pekinensis*, *Cirrhinus molitorella*, and *S.curriculus* were predominant (Supplementary Material S3).

**Figure 2:**
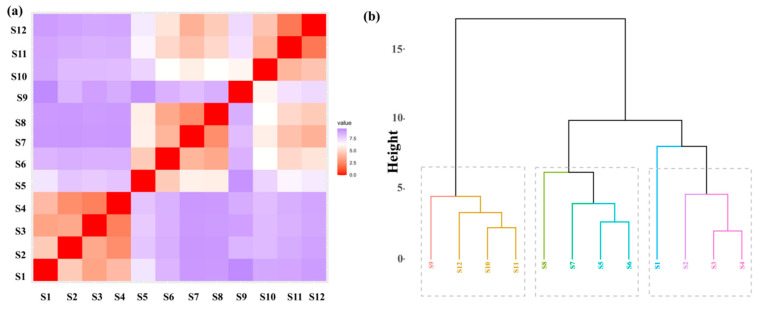
(**a**) Hopkins clustering trend analysis of historical fish species in each reach in the mainstream of the Xijiang River; (**b**) Hierarchical clustering analysis based on the composition of historical fish species in the mainstream of the Xijiang River, the different color boxes show different cluster groups.

After two-score standardization of the historical fish species matrix data at each sampling point along the main stream of the Xijiang River, a Hopkins clustering tendency analysis was conducted. The statistical value was calculated to be 0.7082, indicating that the characteristics of the historical fish species matrix data at the sampling sites are clusterable (Figure 2a). Hierarchical cluster analysis results show that the fish species composition at 12 sampling points along the Xijiang River prior to hydropower development can be clustered into 8 groups (Figure 2b). Groups 1 to 8 consist of fish species compositions from different river segments moving from upstream to downstream: Group 1 comprises the fish species composition at segment S9 (Datengxia Hydropower Station reservoir area); Group 2 includes segments S10 to S12 (from the urban area of Pingle County to Wuzhou City, downstream of the Datengxia Hydropower Station dam site to downstream of the Changzhou Hydropower Station dam site); Group 3 is composed of the fish species at segment S8 (downstream of the Qiaogong Hydropower Station dam site); Group 4 includes segment S7 (downstream of the Letan Hydropower Station dam site); Group 5 consists of segments S5 to S6 (from the Dahua Hydropower Station reservoir area to downstream of the Bailongtan Hydropower Station dam site); Group 6 includes segment S1 (Tianshengqiao First Grade Hydropower Station reservoir area); Group 7 comprises segment S2 (Pingban Hydropower Station dam site reservoir area); and Group 8 includes segments S3 to S4 (from the Longtan Hydropower Station reservoir area to the Yantan Hydropower Station reservoir area). The total number of historical fish species in each cluster group is 129, 114, 131, 116, 125, 52, 88, and 107, respectively.

### 3.2. Selection of Sensitive Indicators

#### 3.2.1. Redundancy Analysis

**Figure 3:**
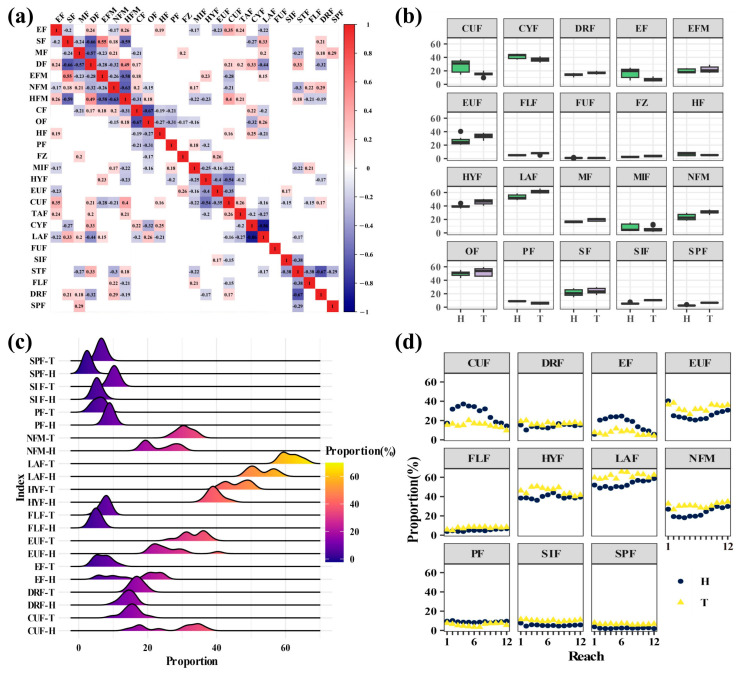
(**a**) Spearman correlations for 25 biological traits; (**b**) Discriminative power screening based on boxplot; (**c**) The concentrated change trend of the historical and current monitoring values of the 11 indicators; (**d**) The sensitivity analysis of the historical reference values and the current monitoring values of 11 indicators. Note: EF, endemic fishes; SF, surface fishes; MF, midwater fishes; DF, demersal fishes; EFM, epistatic fish of mouth; NFM, normotopia fish of mouth; HFM, hypooral fish of mouth; OF, omnivorous fishes; HF, herbivorous fishes; PF, planktivorous fishes; FZ, fishes as zoobenthivores; MIF, migratory fishes; HYF, hydrostatic fishes; EUF, eurytopicity fishes; CUF, current-loving cold water fishes; TAF, tabular fishes; CYF, cylindrical fishes; LAF, lateral fishes; FUF, fusiform fishes; SIF, sinking-egg fishes; FLF, floating egg fish; DRF, drifting-egg fish; SPF, special ways of spawning fish. H, the historical reference values; T, the current monitoring values.

Within 25 ecological habit traits, there are 5 pairs of ecological habit traits that exhibit a high degree of correlation: demersal fish (DF) versus surface fish (SF), with a correlation coefficient (r) of −0.66; hypomouth fish (HFM) versus terminal-mouth fish (NFM), with r = −0.63; omnivorous fish (OF) versus carnivorous fish (CF), with r = −0.67; laterally compressed fish (LAF) versus dorsoventrally flattened fish (TAF), with r = −0.86; and drift egg-producing fish (DRF) versus sticky egg-producing fish (STF), with r = −0.67. Endemic fish (EF) showed lower correlation coefficients with ecological habit traits SF (r = −0.2), NFM (r = −0.17), and LAF (r = −0.22) than with DF (r = 0.24), HFM (r = 0.26), and TAF (r = 0.24). Therefore, three ecological habit traits (DF, HFM, and TAF) were rejected as initial candidate parameters for further assessment (Figure 3a). STF, DRF, OF, and CF had zero correlation coefficients with EF. The indicators DRF and EF, which showed a more significant impact from dam construction on ecological types, were selected for further analysis, while the CF and STF indicators were removed.

#### 3.2.2. Discrimination Power Screening

Based on Redundancy Analysis, 20 ecological habit traits were selected to construct 20 initial indicators for further screening based on discriminative power. The results showed that 11 out of the 20 candidate indicators demonstrated highly significant discriminative ability between historical reference values and current monitoring values, each with an IQ score of 3 (Figure 3b). Consequently, the 9 indicators with weaker discriminative power were removed (CYF, EFM, FUF, FZ, HF, MF, MIF, OF, SF), leaving 11 indicators that can sensitively detect the impact of human disturbances. These remaining indicators are suitable as alternative candidates for further sensitivity analysis. The retained indicators include: endemic fish (EF), terminal-mouth fish (NFM), planktivorous fish (PF), stagnant water fish (HYF), eurytopic fish (EUF), running water fish (CUF), laterally compressed fish (LAF), benthic egg fish (SIF), pelagic egg fish (FLF), drift egg fish (DRF), and special spawning mode fish (SPF)—these 11 ecological percentage indicators.

#### 3.2.3. Sensitivity Analysis

Compared to historical reference values, the trends of the 11 selected candidate indicators varied at each sampling point during the 2022–2023 sampling period (Figure 3c). Among these, the percentage indicators that showed an increasing trend included: special spawning mode fish (SPF), benthic egg fish (SIF), terminal-mouth fish (NFM), laterally compressed fish (LAF), stagnant water fish (HYF), pelagic egg fish (FLF), eurytopic fish (EUF), and drift egg fish (DRF). The percentage indicators that exhibited a decreasing trend were: planktivorous fish (PF), endemic fish (EF), and running water fish (CUF). Notably, EF, EUF, and DRF displayed both increasing and decreasing trends across different river sections. Therefore, for indicators that at some sampling points have values lower than historical data, and at other points have values exceeding historical data, it can be considered that they are insensitive to environmental pressures, as the response of these indicators to environmental stress does not show a clear trend (Figure 3d). Consequently, the remaining sensitive indicators for subsequent scoring include: terminal-mouth fish (NFM), planktivorous fish (PF), stagnant water fish (HYF), running water fish (CUF), laterally compressed fish (LAF), benthic egg fish (SIF), pelagic egg fish (FLF), and special spawning mode fish (SPF).

### 3.3. Expected Values and F-IBI Scores

**Figure 4:**
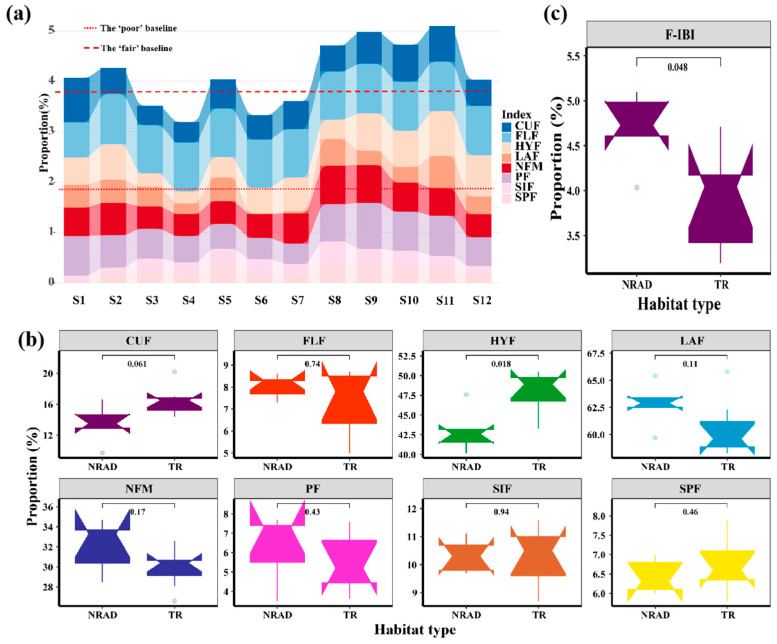
(**a**) F-IBI scores and F-IBI scoring grades for each sampling site; (**b**) Comparison of each sensitive index in two different habitats; (**c**) Comparison of each F-IBI scores in two different habitats. Note: NFM, normotopia fish of mouth; PF, planktivorous fishes; HYF, hydrostatic fishes; CUF, current-loving cold water fishes; LAF, lateral fishes; SIF, sinking-egg fishes; FLF, floating egg fish; SPF, special ways of spawning fish; NRAD, the natural reach between two adjacent dams; TR, transitional region in reservoir.

Based on historical data of fish species composition, the reference value distribution range for each sensitive indicator across eight clustered groups was from 1.9% to 56.8% (Table 2). The expected value distribution ranges for these eight sensitive indicators (NFM, PF, HYF, CUF, LAF, SIF, FLF, SPF) within the eight clusters were 17.2% to 28.4%, 8.0% to 10.2%, 36.2% to 44.0%, 17.3% to 37.1%, 48.6% to 56.8%, 4.5% to 7.7%, 3.8% to 5.6%, and 1.9% to 3.8%, with mean values of 23.0%, 9.1%, 39.4%, 28.1%, 52.4%, 5.3%, 4.8%, and 2.5%, respectively (Table 2). In the current monitoring of 12 river sections, eight sampling points (S1, S2, S5, S8–S12) were rated as ‘Fair’ in terms of F-IBI score, while four sampling points (S3, S4, S6, S7) were rated as ‘Poor’ (Figure 4a). Overall, the health status of the upper and lower reaches was relatively good, while the midstream health status was poorer.

### 3.4. Differences in F-IBI Scores Among Different Habitat Types

Independent sample *t*-tests revealed significant differences in the percentage of lentic fish species (HYF) between different habitat types (NRAD and TR), with a *p*-value of 0.018. However, there were no significant differences in the proportional species numbers of fish with terminal mouths (NFM), planktivorous fish (PF), lotic fish (CUF), laterally compressed fishes (LAF), benthic spawners (SIF), pelagic spawners (FLF), and fish with specialized spawning methods (SPF) between the river sections with dams and reservoirs (NRAD) and the transition zones between dams and reservoirs (TR), with all *p*-values being greater than 0.05 (Figure 4b).

Independent sample *t*-tests demonstrated that the F-IBI (Fish Index of Biotic Integrity) scores at sampling points on the riverine reaches between dams and reservoirs (NRAD) ranged from 4.03 to 5.10, with an average of 4.69. Conversely, the F-IBI scores at sampling points within the transition zone of the reservoir (TR) ranged from 3.19 to 4.71, with an average of 3.88 (Figure 4c). The independent sample *t*-tests revealed significant differences in the average F-IBI scores between the different habitat types (NRAD and TR), with a *p*-value of 0.048.

### 3.5. Identifying the Cumulative Impact of Reservoir Storage Operations on Fish Communities Based on a Relational Model

#### 3.5.1. Relationship Between Variations in F-IBI Total Scores and Environmental Factors at Different Sampling Sites

**Figure 5:**
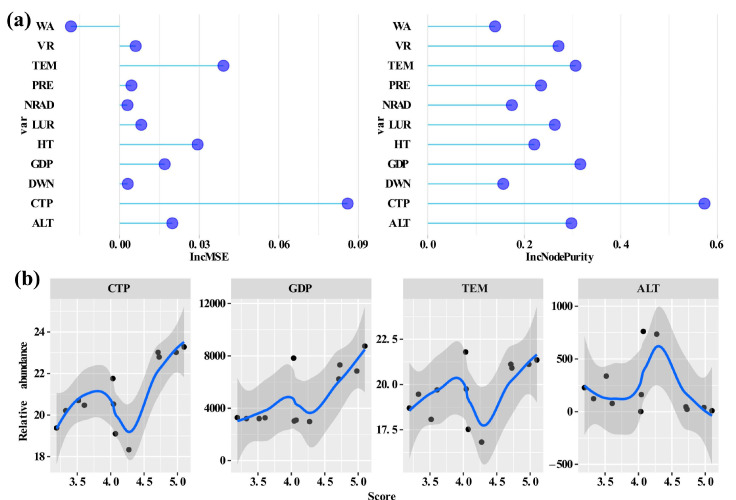
(**a**) Relative contribution of environmental variables based on two different methods (%IncMSE and IncNodePurity); (**b**) Relationships between F-IBI scores and four important environment variables. Note: WA, water Area; VR, average annual runoff; TEM, mean air temperature; PRE, mean annual precipitation; NRAD, natural free-flowing reach between two adjacent dams, LUR, proportion of land use, HT, habitat type, GDP, gross domestic product; DWN, density of water network; CTP, cumulative effect time of power station; ALT, altitude. The shaded area represents the 95% confidence interval.

Results from the Random Forest (RF) model indicate that 11 environmental factors can explain 36.89% of the total variation in F-IBI scores among different sampling sites, with a mean residual of the model being 0.023 (Figure 5a). Four environmental factors—CPT, GDP, TEM, and ALT—ranked in the top four in terms of IncMSE% and IncNodePurity values among all factors, suggesting that these four factors are the main drivers influencing the variability of F-IBI scores across different sampling sites (Figure 5a).

The F-IBI scores at different sampling sites exhibit a roughly linear relationship with the environmental factor GDP, indicating that from upstream to downstream, F-IBI scores generally increase with the rise in GDP. The relationships between the F-IBI scores at different sampling sites and the other three variables can also be well-fitted with polynomial equations (Figure 5b).

#### 3.5.2. The Relationship Between F-IBI Scores for Sensitive Metrics at Different Sampling Sites and Environmental Factors

**Figure 6:**
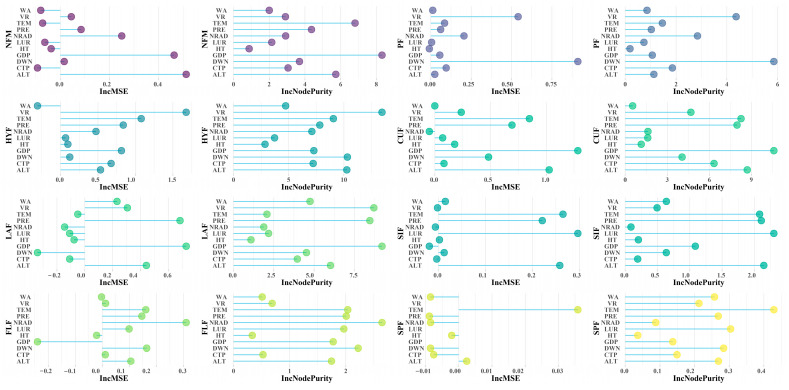
The Random Forest model for predicting F-IBI scores of eight sensitive indicators in 12 reaches of the Xijiang River main stream and the relative contribution ability of environmental variables in predicting F-IBI scores of single sensitive indicators based on IncMSE and IncNodePurity. Note: WA, water area; VR, average annual runoff; TEM, mean air temperature; PRE, mean annual precipitation; NRAD, natural free-flowing reach between two adjacent dams; LUR, proportion of land use; HT, habitat type; GDP, gross domestic product; DWN, density of water network, CTP, cumulative effect time of power station; ALT, altitude. NFM, normotopia fish of mouth; PF, planktivorous fishes; HYF, hydrostatic fishes; CUF, current-loving cold water fishes; LAF, lateral fishes; SIF, sinking-egg fishes; FLF, floating egg fish; SPF, special ways of spawning fish.

In the Random Forest model, 11 environmental factors explain 38.75% (%NFM), 64.52% (%PF), 50.82% (%HYF), 56.31% (%CUF), 3.35% (%LAF), 51.52% (%SIF), 47.3% (%FIF), and 49.91% (%SPF) of the variation in F-IBI scores across different sampling points for eight metrics. Human pressure variables GDP and CPT play the most decisive role in predicting the scores for %NFM, %PF, %HYF, and %CUF; while the human pressure variable CPT is the main factor affecting the variation in %SPF scores among different sampling sites, and GDP is the main factor affecting the variation in %FLF scores; %LAF is most closely related to the environmental filtering variables VR and PRE (Figure 6).

## 4. Discussion

The current investigation identified 139 fish species in the main stream of the Xijiang River, including 120 native species and 19 alien species. The fish fauna is dominated by Cypriniformes (Cyprinidae) and Siluriformes (Cobitidae and Bagridae), consistent with the characteristics of the Pearl River basin. However, historical data reveals significant changes: native species decreased from 168 in the 1980s to 120 currently, while alien species increased. Notably, 30 historically recorded native species were not collected, and several rare species like *Acipenser sinensis*, *Dasyatis akajei*, and *Tenualosa reevesii* have disappeared for years [22,23]. In contrast, eurytopic species increased from 57.1% to 69.7%, while species adapted to fast-flowing habitats decreased from 58.3% to 49.6%. Newly recorded alien species include *Polyodon spathula*, *Prochilodus lineatus*, *Piaractus brachypomus*, *Clarias gariepinus*, *Oreochromis galliaeus*, and *Parachromis managuensis*. *Oreochromis niloticus* and *Coptodon zillii* have become dominant in the upper reservoir area. These changes are attributed to the following: (1) cascade hydropower development, which altered aquatic habitats and decreased fish integrity indices [27]; and (2) aquaculture expansion, with introduced species potentially threatening native fish through competition and predation [22,23].

### 4.1. Selection of Sensitive Metrics

In order to effectively assess environmental stress, an ideal biological indicator should be capable of making precise and sensitive quantitative responses to changes in any environmental stress gradient [34,35]. In light of this, identifying biological indicators that are sensitive to changes in environmental stress is crucial. Numerous studies have shown that visual quantification of biological indicators with abiotic factor data helps to accurately delineate changes in environmental stress [10]. Such comparatively simple statistical methods include the use of scatter plots and multidimensional scaling analysis [36]. However, given the complexity of interactions between environmental stresses, some indicators that signify environmental stress may have complex nonlinear relationships with abiotic parameters, which could lead to inaccurate mapping of actual environmental stress changes [37]. This is especially true in situations where data availability is limited, and when all sampling sites are subjected to extreme disturbances from human activities, making it difficult to determine reference values based on current monitoring, which may result in an inaccurate assessment of environmental stress changes or only minor observed differences in environmental stress variations between sampling sites [38].

The monitoring sites in this study are all located between the eleven cascade hydropower stations on the main stream of the Xijiang River in Guangxi. Compared to before the construction of the dams, the environmental stress (influence of human pressure and environmental filtering) on the fish community structure within the study area has had a more severe impact [39,40]. In this series of environmental stress changes, the sensitive metrics at all sampling sites should show the same trend of change (i.e., the current values of the sensitive metrics at all sampling sites, relative to the reference values, will show synchronous increases or decreases due to the similar environmental stress). The study results indicate that with the completion of the cascade dams on the Xijiang River, the eight key sensitive metrics consistently showed a declining trend at all sampling points, revealing that these indicators are highly sensitive to environmental stress. Among these sensitive metrics, lentic fish species (%HYF) and lotic fish species (%CUF) are particularly sensitive to disturbances from human activities, as has been applied in aquatic biological integrity assessments in multiple regions [16,41]. Previous research has confirmed that reservoirs formed after river damming have a significant impact on the spatial proportion and population numbers of lentic and lotic fish species.

### 4.2. Biological Integrity

Fishes, as a vital component of aquatic ecosystems, serve as indicators of environmental change through their community structure. In the study, the distribution of F-IBI (Fish-based Index of Biotic Integrity) scores among different sampling sites exhibited a serrated pattern, which is largely associated with the alternation between reservoirs and natural flowing river segments along the upstream, midstream, and downstream sections of the river. This distribution characteristic is consistent with the concept of river continuity interruption, suggesting that lake and reservoir habitats may affect the longitudinal continuity of fish assemblages along rivers [42,43]. Researchers have noted that the fish community structure in the natural flowing river segments downstream of power stations is less affected by upstream dams if the river segment is sufficiently long, and it can almost maintain the original regional fish species diversity as it was before the construction of the upstream dam [44,45]. This underscores the importance of maintaining natural flowing habitats between reservoirs, a finding that aligns with studies in other river systems, such as the Yangtze River, where similar patterns of longitudinal connectivity loss were observed due to cascade hydropower development [46]. Furthermore, Lyons found in his research [47] that maintaining a certain length of continuous natural flowing river segments (over 60 km) results in higher scores of the fish integrity index compared to segments where the habitat is severely disrupted (for example, where natural flowing river segments below dams are short or non-existent). This phenomenon has also been documented in the Mekong River, where shorter natural flowing segments between dams exhibited significantly lower fish diversity and altered community structure [48].

Given that the natural flowing habitats between cascade power station reservoirs exhibit higher environmental variability than the transitional zones at the tail of the reservoirs, the average F-IBI score of the natural flowing habitats in this study was significantly higher than that of the transitional zones, confirming the first hypothesis proposed in this study—that river segments with perennial flow between cascade power stations are more beneficial to fish biological integrity compared to transitional zones at the tail of reservoirs. Additionally, we observed that the riverine ecological environment between adjacent power stations is affected to varying degrees. This variability is consistent with findings from other river systems, such as the Columbia River in the United States, where the length and quality of natural flowing segments were found to be critical for maintaining fish populations [49]. The results show that although some river segments (e.g., the Longtan reservoir section at S3) exceed 100 km in natural flow length, their F-IBI score rating is at a ‘poor’ level. This indicates that even if natural connectivity is preserved between cascade power stations, it is difficult to ensure the intrinsic integrity and richness of fish communities due to various influences such as environmental pressures. This phenomenon may be primarily caused by the following factors: firstly, the construction and operation of cascade hydropower inevitably change environmental filtering factors such as downstream hydrology, which affects the distribution of habitats suitable for fish, thereby impacting fish integrity [16,41]; these hydrological changes have been similarly reported in the Amazon River basin, where altered flow regimes led to significant shifts in fish community composition [50]. Secondly, some migratory fish species need to migrate between different river segments to complete their life cycles, and these migration routes are likely to be affected by human pressures (such as the obstruction of continuous construction of hydraulic projects and overfishing) [39,40]. This aligns with findings from the Danube River, where migratory fish populations declined dramatically due to habitat fragmentation and barriers to migration [51]. Moreover, our results are supported by the broader ecological theory on habitat fragmentation, which emphasizes the importance of maintaining not only physical connectivity but also the quality of ecological habitats to sustain fish populations [52].

The classification of F-IBI scores into categories (‘Excellent’, ‘Good’, ‘Fair’, ‘Poor’, and ‘Very Poor’) enables policymakers to prioritize conservation efforts, particularly for river segments with ‘Fair’, ‘Poor’, or ‘Very Poor’ ratings. This framework supports adaptive management strategies balancing hydropower development with biodiversity conservation by maintaining natural flowing river segments as ecological corridors. Such measures ensure migratory fish movement and regional fish diversity, emphasizing ecological integration in hydropower planning for sustainable aquatic ecosystems [11].

### 4.3. Impact of Cascade Development on Fish Community Structure

The Random Forest model indicates that the variability in F-IBI scores at different sampling points is mainly influenced by two environmental factors (CPT and GDP), suggesting that human pressures resulting from cascade hydropower development play a more significant role than hydrological environmental filtering in affecting the biological integrity of fish between reservoirs and in the tailwater transition zones. This supports the second hypothesis. These results are similar to those obtained from the Min River in the Chengdu Plain [53] and in the European Union [54], where the health level of rivers represented by fish biological integrity is influenced not only by the construction of dams but also by major constraining factors such as GDP growth. The study shows that environmental indicators reflecting human pressure, such as GDP and CPT, are the main determinants affecting the scores of most sensitive indicators like F-IBI, indicating that the F-IBI can effectively identify the cumulative impact effects of human pressures after the operation of cascade reservoirs, thereby proving the third hypothesis. Nevertheless, compared to the LAF indicator, NFM, PF, HYF, CUF, FLF, SIF, and SPF have stronger correlations with environmental factors. This may mean that fish with feeding habits such as planktivorous, lentic, lotic, demersal spawning, and those with specialized spawning methods are more susceptible to anthropogenic pressures such as the operation of cascade reservoirs. The weaker correlation of %LAF with environmental factors might be due to the mechanisms driving the variation in species morphological traits being different at different ecological scales, where interspecific trait variability is influenced by evolution, physiology, and diverse life histories [55].

Human pressures, such as the impoundment of cascade power stations acting as ecological filters affecting fish life history strategies, prompt fish with different life history traits to exhibit different adaptive characteristics to the operation of cascade power stations. The laterally compressed fish species distributed in the main stream of the Xijiang River are mainly composed of exotic and indigenous species [56]. Due to the differences in life history strategies among different laterally compressed fish, their responses to human pressures vary. Human pressures, such as cascade power stations, by altering channelized river environments, transform flowing water into standing water, increasing the risk of extinction for native lotic fish species and the invasion of exotic species [40,57]. Moreover, most morphological trait variability in fish occurs at the family level or higher, meaning that the trait-environment relationships among genera and families become more complex [55]. Species turnover after cascade development often obscures most other sources of trait variability. If a functional interpretation of an absolute trait value is consistent across the entire community, then species turnover will produce a strong trait-environment signal, and vice versa [55]. Therefore, before using this indicator, it is necessary to clarify the specific life history characteristics and phylogenetic information of each morphotype of fish.

### 4.4. Recommendations for the Conservation of Fisheries Resources

To address the cumulative impacts of cascade hydropower development and human activities on fish diversity in the Xijiang River, a multifaceted approach to conservation is essential [58]. Our findings highlight two critical stress factors: (1) the temporal effects of hydropower stations, which disrupt longitudinal hydrological connectivity, and (2) the GDP index of river sections, which reflects the intensity of human pressures on fish communities. To mitigate these impacts, we propose the following integrated strategies [59]:

Restoring River Connectivity: Installing fish passage facilities is crucial to restore migratory routes for fish species. While this measure has been implemented on some dams, significant gaps remain, particularly in the Hongshui River tributary, where no fish passages currently exist.

Ecosystem-Based Management: Adopting the principle of ‘ecology first, comprehensive planning, rational utilization, and safeguarding ecological redlines’, we recommend prioritizing the protection of flowing water habitats between reservoirs. This should be complemented by stock enhancement, ecological dispatch, and fisheries supervision to establish a comprehensive protection system.

Long-Term Monitoring and Adaptive Management: Implementing long-term aquatic ecosystem monitoring is essential to assess the effectiveness of conservation measures and adapt strategies accordingly. This approach ensures continuous improvement in fisheries management and resource protection.

Addressing Multiple Stressors: Beyond hydropower impacts, other factors such as sand mining, commercial navigation, urban development, and agriculture exert significant pressure on fish diversity. Policy initiatives should aim to mitigate these threats through coordinated, multi-sectoral efforts.

By integrating these strategies, we aim to balance hydropower development with biodiversity conservation, ensuring the sustainable use of fishery resources in the Xijiang River basin.

## 5. Conclusions

Through redundancy, discrimination, and sensitivity analyses of biotic integrity indices, eight key metrics, including buccal position fish, plankton-feeding fish, lentic fish, lotic fish, laterally compressed fish, benthic egg-laying fish, pelagic egg-laying fish, and fish with specialized spawning behaviors, were identified to sensitively detect anthropogenic disturbances in the natural flowing river sections and reservoir transition zones of the mainstream Xijiang River. These metrics were utilized to construct a Fish-based Index of Biotic Integrity (F-IBI) tailored to different river segments within the Xijiang mainstream. Applying this F-IBI framework to assess river health across 12 sampling sites revealed that upstream and downstream sections maintained relatively good health, whereas midstream sections exhibited poorer conditions. Additionally, F-IBI scores in reservoir transition zones were significantly lower than those in the natural flowing sections between reservoirs. Utilizing Random Forest models, the F-IBI effectively identified the cumulative impacts of cascade hydropower development on fish community structure, highlighting the critical roles of cumulative temporal effects and the GDP index of river segments. To effectively conserve fish resources in the Xijiang mainstream, the study recommends prioritizing the protection of natural flowing river habitats between reservoirs and developing environmental regulatory policies tailored to the degree of human development in each river segment.

## Figures and Tables

**Table 1:** Classification and introduction of study sites based on habitat characters.

Sampling Site	Habitat Type		The Length of Natural Lotic Reach Between Dams (km)
	Natural Free-Flowing Reach of Dams (NRAD)	Transitional Region in Reservoir (TR)	
S1		√	81.0
S2		√	17.0
S3	√		106
S4		√	27.0
S5		√	6.0
S6		√	23.0
S7		√	7.0
S8	√		110
S9	√		110
S10	√		56.0
S11	√		56.0
S12	√		350

**Table 2:** Reference values of each sensitive metric (%) in the eight cluster groups.

Metrics (%)	S1	S2	S3~S4	S5~S6	S7	S8	S9	S10~S12
NFM	26.9	19.3	17.2	19.7	20.7	24.4	27.2	28.4
PF	9.6	10.2	8.9	8.5	8.6	9.2	8.0	9.6
HYF	38.5	38.6	36.2	40.2	44.0	40.5	38.4	38.5
CUF	17.3	31.8	37.1	35.0	30.2	32.1	23.2	18.4
LAF	51.9	48.8	48.6	50	51.7	55.0	56.8	56.1
SIF	7.7	4.5	5.7	4.9	5.2	4.6	4.8	5.3
FLF	3.8	4.5	3.8	4.9	5.2	4.6	5.6	6.1
SPF	3.8	2.3	1.9	2.5	2.6	2.3	2.4	1.9

Note: NFM, normotopia fish of mouth; PF, planktivorous fishes; HYF, hydrostatic fishes; CUF, current-loving cold water fishes; LAF, lateral fishes; SIF, sinking-egg fishes; FLF, floating egg fish; SPF, special ways of spawning fish.

## Data Availability

Data will be available upon request.

## Supplementary Materials

The following supporting information can be downloaded at: https://www.mdpi.com/article/10.3390/ani15040495/s1, S1: Changes in the Water Surface Area of the Eleven-Level Cascade Hydropower Stations in the mainstream of the Xijiang River; S2: Historical species composition of fish of the mainstream of the Xijiang River. S3: The complete list of fish species in 2022–2023 of the mainstream of the Xijiang River.
