# Supplementary material for: Cumulative Ecological Impact of Cascade Hydropower Development on Fish Community Structure in the Main Stream of the Xijiang River, China
Source: Animals (Basel). 2025 Feb 10;15(4):495. doi: 10.3390/ani15040495 (PMC11851437; doi:10.3390/ani15040495)
Supplement: Supplementary file 1 [file animals-15-00495-s001.zip › animals-3433716-supplementary.pdf]

Supplementary Material

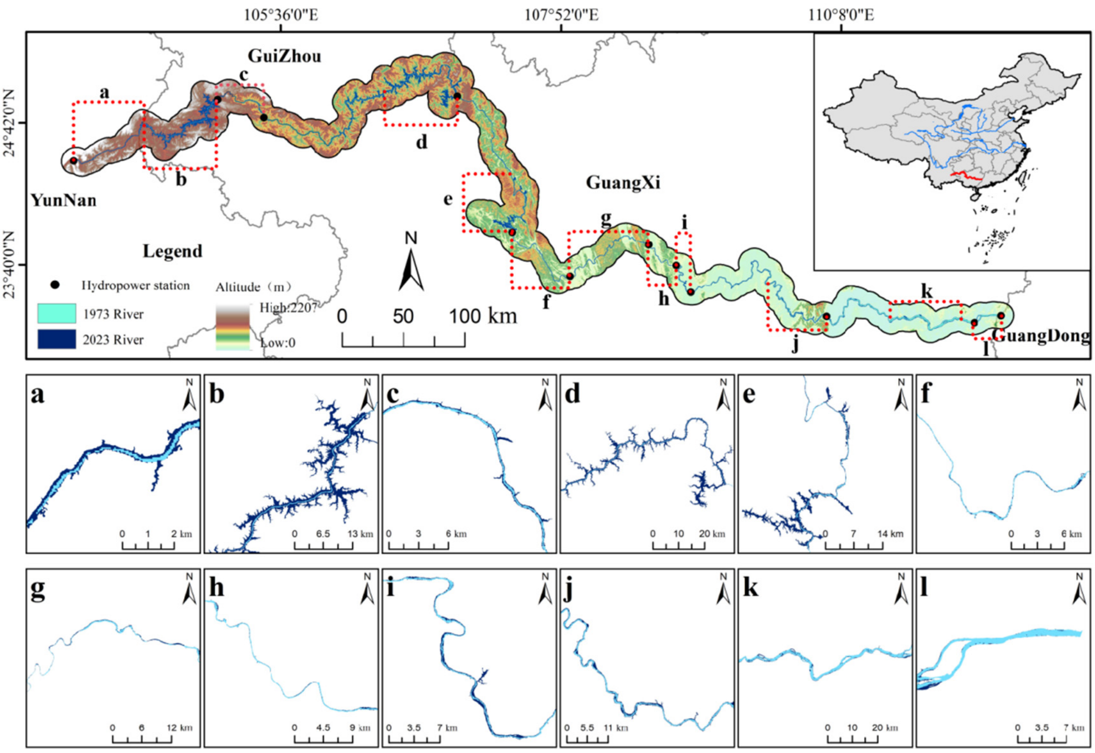

S1 Changes in the Water Surface Area of the Eleven-Level Cascade Hydropower Stations in the mainstream of the Xijiang River

S2 Historical species composition of fish of the mainstream of the Xijiang River

species	EF	SF	MF	DF	EFM	NFM	HFM	CF	OF	HF	PF	FZ	MIF	HYF	EUF	CUF	TAF	CYF	LAF	FUF	SIF	STF	FLF	DRF	SPF
Hemitrygon akajei	0	0	0	1	0	0	1	1	0	0	0	0	1	0	0	0	1	0	0	0	0	0	0	0	1
Acipenser sinensis	0	0	0	1	0	0	1	1	0	0	0	0	1	0	0	0	0	1	0	0	1	0	0	0	0
Tenualosa reevesii	0	1	0	0	0	1	0	0	1	0	0	0	1	0	0	0	0	0	1	0	1	0	0	0	0
Clupanodon thrissa	0	0	1	0	0	1	0	0	0	0	1	0	1	0	0	0	0	0	1	0	0	1	0	0	0
Konosirus punctatus	0	0	1	0	0	1	0	0	0	0	1	0	1	0	0	0	0	0	1	0	0	1	0	0	0
Coilia grayi	0	0	1	0	0	0	1	0	0	0	1	0	1	0	0	0	0	0	1	0	0	0	1	0	0
Anguilla japonica	0	0	0	1	1	0	0	1	0	0	0	0	1	0	0	0	0	1	0	0	0	0	1	0	0
Anguilla marmorata	0	0	0	1	1	0	0	1	0	0	0	0	1	0	0	0	0	1	0	0	0	0	1	0	0
Salanx chinensis	0	1	0	0	1	0	0	0	1	0	0	0	1	0	0	0	0	1	0	0	0	1	0	0	0
Traccatichthys pulcher	0	0	0	1	0	0	1	0	1	0	0	0	0	1	0	0	0	0	0	1	0	1	0	0	0
Oreonectes platycephalus	1	0	0	1	0	0	1	0	1	0	0	0	0	1	0	0	0	0	0	1	0	1	0	0	0
Schistura incerta	1	0	0	1	0	0	1	0	1	0	0	0	0	1	0	0	0	0	1	0	0	1	0	0	0
Schistura fasciolatus	0	0	0	1	0	0	1	0	1	0	0	0	0	1	0	0	0	0	1	0	0	1	0	0	0
Sinibotia robusta	0	0	0	1	0	0	1	0	1	0	0	0	0	1	0	0	0	0	1	0	0	1	0	0	0
Sinibotia pulchra	0	0	0	1	0	0	1	0	1	0	0	0	0	1	0	0	0	0	1	0	0	1	0	0	0
Parabotia fasciata	0	0	0	1	0	0	1	0	1	0	0	0	0	1	0	0	0	0	1	0	0	1	0	0	0
Parabotia maculosus	0	0	0	1	0	0	1	0	1	0	0	0	0	1	0	0	0	0	1	0	0	1	0	0	0
Leptobotia pellegrini	0	0	0	1	0	0	1	0	1	0	0	0	0	1	0	0	0	0	1	0	0	0	0	1	0
Cobitis sinensis	0	0	0	1	0	0	1	0	1	0	0	0	0	1	0	0	0	0	1	0	1	0	0	0	0
Cobitis arenae	1	0	0	1	0	0	1	0	1	0	0	0	0	1	0	0	0	0	1	0	1	0	0	0	0
Misgurnus anguillicaudatus	0	0	0	1	0	0	1	0	1	0	0	0	0	1	0	0	0	1	0	0	0	1	0	0	0
Paramisgurnus dabryanus	0	0	0	1	0	0	1	0	1	0	0	0	0	1	0	0	0	0	1	0	0	1	0	0	0
Zacco acanthogenys	0	0	1	0	0	1	0	0	1	0	0	0	0	0	1	0	0	0	1	0	1	0	0	0	0
Opsariichthys bidens	0	1	0	0	1	0	0	0	1	0	0	0	0	0	1	0	0	0	1	0	1	0	0	0	0
Aphyocypris arcus	1	0	1	0	0	1	0	0	1	0	0	0	0	0	1	0	0	0	1	0	0	1	0	0	0
Rasbora steineri	0	1	0	0	1	0	0	0	1	0	0	0	0	1	0	0	0	0	1	0	0	1	0	0	0
Mylopharyngodon piceus	0	0	0	1	0	1	0	1	0	0	0	0	1	0	0	0	0	1	0	0	0	0	0	1	0
Ctenopharyngodon idella	0	0	0	1	0	1	0	0	0	1	0	0	1	0	0	0	0	1	0	0	0	0	0	1	0
Squaliobarbus curriculus	0	0	1	0	0	1	0	0	1	0	0	0	0	0	1	0	0	1	0	0	0	0	0	1	0
Ochetobius elongatus	0	0	1	0	0	1	0	0	1	0	0	0	1	0	0	0	0	0	1	0	0	0	0	1	0
Elopichthys bambusa	0	1	0	0	0	1	0	1	0	0	0	0	0	0	0	1	0	1	0	0	0	0	0	1	0
Metzia lineata	0	1	0	0	1	0	0	0	1	0	0	0	0	0	1	0	0	0	1	0	0	1	0	0	0
Metzia formosae	0	1	0	0	1	0	0	0	1	0	0	0	0	0	1	0	0	0	1	0	0	1	0	0	0
Sinibrama macrops	0	1	0	0	0	1	0	0	1	0	0	0	0	0	1	0	0	0	1	0	0	1	0	0	0
Sinibrama melrosei	0	1	0	0	0	1	0	0	1	0	0	0	0	0	1	0	0	0	1	0	0	1	0	0	0
Ancherythroculter lini	0	1	0	0	1	0	0	0	1	0	0	0	0	0	1	0	0	0	1	0	0	1	0	0	0
Pseudolaubuca sinensis	0	1	0	0	1	0	0	0	1	0	0	0	0	1	0	0	0	0	1	0	0	0	0	1	0
Pseudolaubuca engraulis	0	1	0	0	1	0	0	0	1	0	0	0	0	1	0	0	0	0	1	0	0	0	0	1	0
Toxabramis houdemeri	0	1	0	0	1	0	0	0	1	0	0	0	0	1	0	0	0	0	1	0	0	1	0	0	0
Toxabramis hoffmanni	1	1	0	0	1	0	0	0	1	0	0	0	0	1	0	0	0	0	1	0	0	1	0	0	0
Hemiculter leucisculus	0	1	0	0	1	0	0	0	1	0	0	0	0	1	0	0	0	0	1	0	0	1	0	0	0

species	EF	SF	MF	DF	EFM	NFM	HFM	CF	OF	HF	PF	FZ	MIF	HYF	EUF	CUF	TAF	CYF	LAF	FUF	SIF	STF	FLF	DRF	SPF
Hemiculterella wui	0	1	0	0	1	0	0	0	1	0	0	0	0	1	0	0	0	0	1	0	0	1	0	0	0
Pseudohemiculter dispar	0	1	0	0	1	0	0	0	1	0	0	0	0	1	0	0	0	0	1	0	0	1	0	0	0
Pseudohemiculter hainanensis	0	1	0	0	1	0	0	0	1	0	0	0	0	1	0	0	0	0	1	0	0	1	0	0	0
Culter alburnus	0	1	0	0	1	0	0	1	0	0	0	0	0	1	0	0	0	0	1	0	0	1	0	0	0
Culter recurviceps	0	1	0	0	1	0	0	1	0	0	0	0	0	1	0	0	0	0	1	0	0	1	0	0	0
Chanodichthys mongolicus	0	1	0	0	1	0	0	1	0	0	0	0	0	1	0	0	0	0	1	0	0	1	0	0	0
Chanodichthys erythropterus	0	1	0	0	1	0	0	1	0	0	0	0	0	1	0	0	0	0	1	0	0	1	0	0	0
Parabramis pekinensis	0	0	1	0	0	1	0	0	0	1	0	0	0	1	0	0	0	0	1	0	0	0	0	1	0
Megalobrama terminalis	0	0	1	0	0	1	0	0	1	0	0	0	0	0	1	0	0	0	1	0	0	1	0	0	0
Distoechodon tumirostris	0	0	1	0	0	0	1	0	0	0	0	1	0	0	1	0	0	0	1	0	0	1	0	0	0
Xenocypris argentea	0	0	1	0	0	0	1	0	0	0	0	1	0	0	1	0	0	0	1	0	0	0	0	1	0
Xenocypris davidi	0	0	1	0	0	0	1	0	0	0	0	1	0	0	1	0	0	0	1	0	0	1	0	0	0
Hypophthalmichthys molitrix	0	1	0	0	0	1	0	0	0	0	1	0	0	0	1	0	0	0	1	0	0	0	0	1	0
Aristichthys nobilis	0	1	0	0	0	1	0	0	0	0	1	0	0	0	1	0	0	0	1	0	0	0	0	1	0
Hemibarbus labeo	0	0	0	1	0	0	1	0	1	0	0	0	0	0	1	0	0	1	0	0	0	1	0	0	0
Hemibarbus medius	0	0	0	1	0	0	1	0	1	0	0	0	0	0	1	0	0	1	0	0	0	1	0	0	0
Hemibarbus maculatus	0	0	0	1	0	0	1	0	1	0	0	0	0	0	1	0	0	1	0	0	0	1	0	0	0
Pseudorasbora parva	0	1	0	0	0	1	0	0	1	0	0	0	0	1	0	0	0	0	1	0	0	1	0	0	0
Sarcocheilichthys Parvus	0	0	0	1	0	0	1	0	0	0	1	0	0	0	0	1	0	0	1	0	0	1	0	0	0
Sarcocheilichthys nigripinnis	0	0	1	0	0	0	1	0	1	0	0	0	0	1	0	0	0	0	1	0	0	1	0	0	0
Squalidus argentatus	0	0	1	0	0	0	1	0	1	0	0	0	0	0	1	0	0	1	0	0	0	1	0	0	0
Abbottina rivularis	0	0	0	1	0	0	1	0	1	0	0	0	0	0	1	0	0	1	0	0	1	0	0	0	0
Microphysogobio elongate	0	0	0	1	0	0	1	0	1	0	0	0	0	1	0	0	0	1	0	0	0	1	0	0	0
Microphysogobio kiatingensis	0	0	0	1	0	0	1	0	1	0	0	0	0	1	0	0	0	1	0	0	0	1	0	0	0
Saurogobio dabryi	0	0	0	1	0	0	1	0	1	0	0	0	0	0	0	1	0	1	0	0	0	0	0	1	0
Gobiobotia kolleri	0	0	0	1	0	0	1	0	1	0	0	0	0	0	0	1	0	1	0	0	0	0	0	1	0
Acheilognathus barbatus	1	0	1	0	0	0	1	0	1	0	0	0	0	1	0	0	0	0	1	0	0	0	0	0	1
Acheilognathus tonkinensis	1	0	1	0	0	0	1	0	1	0	0	0	0	1	0	0	0	0	1	0	0	0	0	0	1
Rhodeus ocellatus	0	0	1	0	0	0	1	0	1	0	0	0	0	1	0	0	0	0	1	0	0	0	0	0	1
Rhodeus spinalis	0	0	1	0	0	0	1	0	1	0	0	0	0	1	0	0	0	0	1	0	0	0	0	0	1
Puntius semifasciolatus	0	0	1	0	0	0	1	0	1	0	0	0	0	1	0	0	0	0	1	0	0	1	0	0	0
Spinibarbus caldwelli	0	0	1	0	0	0	1	0	0	1	0	0	0	0	0	1	0	1	0	0	0	1	0	0	0
Spinibarbus denticulatus	0	0	1	0	0	0	1	0	0	1	0	0	0	0	0	1	0	1	0	0	0	1	0	0	0
Luciocyprinus langsoni	1	1	0	0	0	1	0	1	0	0	0	0	0	0	0	1	0	1	0	0	0	0	0	1	0
Acrossocheilus parallens	1	0	1	0	0	0	1	0	1	0	0	0	0	0	0	1	0	0	1	0	0	0	0	1	0
Acrossocheilus kreyenbergii	0	0	1	0	0	0	1	0	1	0	0	0	0	0	0	1	0	0	1	0	0	0	0	1	0
Acrossocheilus iridescens	0	0	1	0	0	0	1	0	1	0	0	0	0	0	0	1	0	0	1	0	0	0	0	1	0
Acrossocheilus elongatus	0	0	1	0	0	0	1	0	1	0	0	0	0	0	0	1	0	0	1	0	0	0	0	1	0
Acrossocheilus yunnanensis	0	0	0	1	0	0	1	0	1	0	0	0	0	0	0	1	0	0	1	0	0	1	0	0	0
Acrossocheilus clivosius	0	0	0	1	0	0	1	0	1	0	0	0	0	0	0	1	0	0	1	0	0	1	0	0	0

species	EF	SF	MF	DF	EFM	NFM	HFM	CF	OF	HF	PF	FZ	MIF	HYF	EUF	CUF	TAF	CYF	LAF	FUF	SIF	STF	FLF	DRF	SPF
Onychostoma barbatum	0	0	0	1	0	0	1	0	1	0	0	0	0	0	0	1	0	0	1	0	0	1	0	0	0
Onychostoma leptura	0	0	0	1	0	0	1	0	1	0	0	0	0	0	0	1	0	0	1	0	0	1	0	0	0
Onychostoma gerlachi	0	0	0	1	0	0	1	0	1	0	0	0	0	0	0	1	0	0	1	0	0	1	0	0	0
Onychostoma lini	0	0	0	1	0	0	1	0	1	0	0	0	0	0	0	1	0	0	1	0	0	1	0	0	0
Onychostoma ovalis rhomboides	1	0	0	1	0	0	1	0	1	0	0	0	0	0	0	1	0	0	1	0	0	1	0	0	0
Onychostoma rarum	0	0	0	1	0	0	1	0	1	0	0	0	0	0	0	1	0	0	1	0	0	1	0	0	0
Tor brevifilis	0	0	0	1	0	0	1	1	0	0	0	0	0	0	0	1	0	0	1	0	0	1	0	0	0
Tor zonatus	0	0	0	1	0	0	1	1	0	0	0	0	0	0	0	1	0	0	1	0	0	1	0	0	0
Bangana discognathoides	1	0	0	1	0	0	1	0	1	0	0	0	0	0	0	1	0	0	1	0	0	1	0	0	0
Decorus decora	1	0	0	1	0	0	1	0	1	0	0	0	0	0	0	1	0	0	1	0	0	1	0	0	0
Cirrhinus molitorella	0	0	0	1	0	0	1	0	0	1	0	0	0	0	1	0	0	0	1	0	0	1	0	0	0
Osteochilus salsburyi	0	0	0	1	0	0	1	1	0	0	0	0	0	1	0	0	0	0	1	0	0	1	0	0	0
Rectoris posehensis	1	0	0	1	0	0	1	0	0	1	0	0	0	1	0	0	0	1	0	0	0	1	0	0	0
Pseudocrossocheilus bamaensis	1	0	0	1	0	0	1	0	0	1	0	0	0	1	0	0	0	1	0	0	0	1	0	0	0
Parasinilabeo longiventralis	1	0	0	1	0	0	1	0	0	1	0	0	0	0	0	1	0	1	0	0	0	1	0	0	0
Semilabeo notabilis	1	0	0	1	0	0	1	0	0	1	0	0	0	0	0	1	0	1	0	0	0	1	0	0	0
Semilabeo obscurus	1	0	0	1	0	0	1	0	0	1	0	0	0	0	0	1	0	1	0	0	0	1	0	0	0
Pseudogyrinocheilus prochilus	0	0	0	1	0	0	1	0	0	1	0	0	0	0	0	1	0	1	0	0	0	1	0	0	0
Ptychidio jordani	1	0	0	1	0	0	1	0	1	0	0	0	0	1	0	0	0	1	0	0	0	1	0	0	0
Ptychidio longibarbus	1	0	0	1	0	0	1	0	1	0	0	0	0	1	0	0	0	1	0	0	0	1	0	0	0
Hongshuia microstomatus	1	0	0	1	0	0	1	0	0	1	0	0	0	0	0	1	0	1	0	0	0	1	0	0	0
Garra orientalis	0	0	0	1	0	0	1	0	0	0	1	0	0	0	0	1	0	1	0	0	0	1	0	0	0
Discogobio yunnanensis	0	0	0	1	0	0	1	0	0	0	1	0	0	0	0	1	0	1	0	0	0	1	0	0	0
Discogobio laticeps	0	0	0	1	0	0	1	0	0	0	1	0	0	0	0	1	0	1	0	0	0	1	0	0	0
Discogobio multilineatus	1	0	0	1	0	0	1	0	0	0	1	0	0	0	0	1	0	1	0	0	0	1	0	0	0
Discogobio tetrabarbatus	1	0	0	1	0	0	1	0	0	0	1	0	0	0	0	1	0	1	0	0	0	1	0	0	0
Discocheilus wui	1	0	0	1	0	0	1	0	0	0	1	0	0	0	0	1	0	1	0	0	0	1	0	0	0
Procypris merus	1	0	0	1	0	0	1	0	0	0	1	0	0	0	0	1	0	0	1	0	0	1	0	0	0
Cyprinus multitaeniata	0	0	0	1	0	0	1	1	0	0	0	0	0	0	0	1	0	0	1	0	0	1	0	0	0
Cyprinus rubrofuscus	0	0	0	1	0	0	1	0	1	0	0	0	0	0	1	0	0	0	1	0	0	1	0	0	0
Carassioides cantonensis	0	0	0	1	0	0	1	0	1	0	0	0	0	1	0	0	0	0	1	0	0	1	0	0	0
Carassius auratus	0	0	1	0	0	1	0	0	1	0	0	0	0	0	1	0	0	0	1	0	0	1	0	0	0
Vanmanenia pingchowensis	0	0	0	1	0	0	1	0	1	0	0	0	0	0	0	1	0	1	0	0	0	1	0	0	0
Vanmanenia xinyiensis	1	0	0	1	0	0	1	0	1	0	0	0	0	0	0	1	0	1	0	0	0	1	0	0	0
Pseudogastromyzon fangi	1	0	0	1	0	0	1	0	1	0	0	0	0	0	0	1	1	0	0	0	0	1	0	0	0
Paraprotomyzon bamaensis	1	0	0	1	0	0	1	0	1	0	0	0	0	0	0	1	1	0	0	0	0	1	0	0	0
Beaufortia pingi	1	0	0	1	0	0	1	0	1	0	0	0	0	0	0	1	1	0	0	0	0	1	0	0	0
Beaufortia kweichowensis	0	0	0	1	0	0	1	0	1	0	0	0	0	0	0	1	1	0	0	0	0	1	0	0	0
Balitora kwangsiensis	1	0	0	1	0	0	1	0	1	0	0	0	0	0	0	1	1	0	0	0	0	1	0	0	0

species	EF	SF	MF	DF	EFM	NFM	HFM	CF	OF	HF	PF	FZ	MIF	HYF	EUF	CUF	TAF	CYF	LAF	FUF	SIF	STF	FLF	DRF	SPF
Sinogastromyzon wui	1	0	0	1	0	0	1	0	1	0	0	0	0	0	0	1	1	0	0	0	0	1	0	0	0
Pterocryptis anomala	0	0	0	1	1	0	0	0	0	0	0	1	0	0	1	0	0	0	1	0	0	1	0	0	0
Silurus asotus	0	0	0	1	1	0	0	1	0	0	0	0	0	0	1	0	0	0	1	0	0	1	0	0	0
Silurus duanensis	1	0	0	1	1	0	0	1	0	0	0	0	0	1	0	0	0	0	1	0	0	1	0	0	0
Clarias fuscus	0	0	0	1	1	0	0	1	0	0	0	0	0	1	0	0	0	1	0	0	0	1	0	0	0
Cranoglanis bouderius	0	0	0	1	0	0	1	1	0	0	0	0	0	1	0	0	0	0	1	0	0	1	0	0	0
Tachysurus fulvidraco	0	0	0	1	0	0	1	1	0	0	0	0	0	0	1	0	0	0	1	0	1	0	0	0	0
Tachysurus virgatus	0	0	0	1	0	0	1	1	0	0	0	0	0	0	1	0	0	0	1	0	0	1	0	0	0
Tachysurus argentivittatus	0	0	0	1	0	0	1	1	0	0	0	0	0	0	1	0	0	1	0	0	0	1	0	0	0
Tachysurus intermedius	0	0	0	1	0	0	1	1	0	0	0	0	0	0	1	0	0	1	0	0	0	1	0	0	0
Tachysurus vachelli	0	0	0	1	0	0	1	1	0	0	0	0	0	0	1	0	0	1	0	0	0	1	0	0	0
Tachysurus crassilabris	0	0	0	1	0	0	1	1	0	0	0	0	0	0	1	0	0	1	0	0	0	1	0	0	0
Hemibagrus guttatus	0	0	0	1	0	0	1	1	0	0	0	0	0	0	1	0	0	0	1	0	0	1	0	0	0
Hemibagrus macropterus	0	0	0	1	0	0	1	1	0	0	0	0	0	0	1	0	0	0	1	0	0	1	0	0	0
Glyptothorax sinensis	0	0	0	1	0	0	1	1	0	0	0	0	0	0	0	1	1	0	0	0	0	1	0	0	0
Pareuchiloglanis longicauda	1	0	0	1	0	0	1	1	0	0	0	0	0	0	0	1	1	0	0	0	0	1	0	0	0
Liobagrus anguillicauda	1	0	0	1	1	0	0	1	0	0	0	0	0	0	0	1	0	1	0	0	0	1	0	0	0
Xiurenbagrus xiurenensis	1	0	0	1	0	0	1	1	0	0	0	0	0	0	0	1	0	1	0	0	0	1	0	0	0
Xiurenbagrus gigas	1	0	0	1	0	0	1	1	0	0	0	0	0	0	0	1	0	1	0	0	0	1	0	0	0
Mugil cephalus	0	1	0	0	0	0	1	0	1	0	0	0	0	0	0	1	0	1	0	0	0	1	0	0	0
Hyporhamphus intermedius	0	1	0	0	1	0	0	0	0	0	1	0	1	0	0	0	0	1	0	0	0	1	0	0	0
Oryzias latipes	0	1	0	0	1	0	0	0	0	0	1	0	0	1	0	0	0	0	1	0	0	1	0	0	0
Monopterus albus	0	0	0	1	0	1	0	1	0	0	0	0	0	1	0	0	0	1	0	0	0	0	1	0	0
Mastacembelus armatus	0	0	0	1	0	0	1	0	1	0	0	0	0	0	1	0	0	1	0	0	0	1	0	0	0
Lateolabrax japonicus	0	1	0	0	1	0	0	1	0	0	0	0	1	0	0	0	0	0	1	0	0	1	0	0	0
Coreoperca whiteheadi	0	1	0	0	0	1	0	1	0	0	0	0	0	0	0	1	0	0	1	0	0	0	0	1	0
Siniperca undulata	0	1	0	0	1	0	0	1	0	0	0	0	0	0	0	1	0	0	1	0	0	0	0	1	0
Siniperca scherzeri	0	1	0	0	0	1	0	1	0	0	0	0	0	0	0	1	0	0	1	0	0	0	0	1	0
Siniperca knerii	0	1	0	0	0	1	0	1	0	0	0	0	0	0	0	1	0	0	1	0	0	0	0	1	0
Odontobutis sinensis	0	0	0	1	1	0	0	1	0	0	0	0	0	1	0	0	0	1	0	0	0	1	0	0	0
Eleotris oxycephala	0	0	0	1	0	1	0	1	0	0	0	0	0	1	0	0	0	1	0	0	0	1	0	0	0
Philypnus chalmersi	0	0	0	1	1	0	0	1	0	0	0	0	0	1	0	0	0	1	0	0	0	1	0	0	0
Neodontobutis hainanensis	0	0	0	1	1	0	0	1	0	0	0	0	0	1	0	0	0	1	0	0	0	1	0	0	0
Mugilogobius myxodermus	0	0	0	1	0	1	0	1	0	0	0	0	0	1	0	0	0	1	0	0	0	1	0	0	0
Glossogobius giuris	0	0	0	1	1	0	0	1	0	0	0	0	0	1	0	0	0	1	0	0	0	1	0	0	0
Rhinogobius giurinus	0	0	0	1	0	1	0	1	0	0	0	0	0	1	0	0	0	1	0	0	0	1	0	0	0
Rhinogobius duospilus	0	0	0	1	0	1	0	1	0	0	0	0	0	1	0	0	0	1	0	0	0	1	0	0	0
Rhinogobius filamentosus	0	0	0	1	0	1	0	1	0	0	0	0	0	1	0	0	0	1	0	0	0	1	0	0	0
鱼 *Rhinogobius leavelli*	0	0	0	1	0	1	0	1	0	0	0	0	0	1	0	0	0	1	0	0	0	1	0	0	0
Rhinogobius yaoshanensis	1	0	0	1	0	1	0	1	0	0	0	0	0	1	0	0	0	1	0	0	0	1	0	0	0
Anabas testudineus	0	0	0	1	0	1	0	1	0	0	0	0	0	1	0	0	0	0	1	0	0	0	1	0	0
Macropodus opercularis	0	1	0	0	0	1	0	0	1	0	0	0	0	1	0	0	0	0	1	0	0	0	1	0	0

species	EF	SF	MF	DF	EFM	NFM	HFM	CF	OF	HF	PF	FZ	MIF	HYF	EUF	CUF	TAF	CYF	LAF	FUF	SIF	STF	FLF	DRF	SPF
Macropodus ocellatus	0	1	0	0	0	1	0	0	1	0	0	0	0	1	0	0	0	0	1	0	0	0	1	0	0
Channa maculate	0	0	1	0	0	1	0	1	0	0	0	0	0	1	0	0	0	1	0	0	0	0	1	0	0
Channa asiatica	0	0	1	0	0	1	0	1	0	0	0	0	0	1	0	0	0	1	0	0	0	1	0	0	0
Channa gachua	0	0	1	0	0	1	0	1	0	0	0	0	0	1	0	0	0	1	0	0	0	1	0	0	0
Cynoglossus trigrammus	0	0	0	1	0	0	1	0	0	0	0	1	1	0	0	0	1	0	0	0	0	1	0	0	0
Takifugu ocellatus	0	0	0	1	0	1	0	0	1	0	0	0	1	0	0	0	0	1	0	0	0	1	0	0	0

Note: EF-Endemic fishes, SF-Surface fishes, MF-Midwater fishes, DF-Demersal fishes, EFM-Epistatic fish of mouth, NFM-Normotopia fish of mouth, HFM-Hypooral fish of mouth, CF-Carnivorous fishes, OF-Omnivorous fishes, HF-Herbivorous fishes, PF-Planktivorous fishes, FZ-Fishes as zoobenthivores, MIF-Migratory fishes, HYF-Hydrostatic fishes, EUF-Eurytopicity fishes, CUF-Current-loving cold water fishies, TAF-Tabular fishes, CYF-Cylindrical fishes, LAF-Lateral fishes, FUF-Fusiform fishes, SIF-Sinking-egg fishes, STF-Stick-egg fishes, FLF-Floating egg fish, DRF-Drifting-egg fish, SPF-Special ways of spawning fish.

S3 The complete list of fish species in 2022-2023 of the mainstream of the Xijiang River

Species	Sampling points											
	S1	S2	S3	S4	S5	S6	S7	S8	S9	S10	S11	S12
**Acipenseriformes**												
**Polyodontidae**												
1.*Polyodon spathula*△			+	+	+	+	+					
**Salmoniformes**												
**Salangidae**												
2.*Neosalanx taihuensis*△		+	+	+	+	+	+	+	+	+	+	+
**Clupeiformes**												
**Engraulidae**												
3.*Coilia grayi*									+	+	+	+
4.*Clupanodon thrissa*										+	+	+
**Anguilliformes**												
**Anguillidae**												
5.*Anguilla japonica*			+	+	+	+	+	+	+	+	+	+
6.*Anguilla marmorata*					+			+			+	+
**Characiformes**												
**Characidae**												
7.*Prochilodus lineatus*△			+	+	+	+	+	+	+	+	+	+
8.*Piaractus brachypomus*△			+	+	+	+	+					
**Cypriniformes**												
**Cobitidae**												
9.*Micronemacheilus pulcher*			+	+	+	+	+	+	+			
10.*Schistura fasciolata*			+	+	+	+	+	+				
11.*Schistura incerta*					+							
12.*Sinibotia robusta*		+	+	+	+	+	+	+	+	+		
13.*Sinibotia pulchra*			+	+	+			+				
14.*Parabotia fasciatus*								+				
15.*Cobitis sinensis*				+	+	+	+	+				
16.*Misgurnus anguillicaudatus*	+	+	+	+	+	+	+	+	+	+	+	+
17.*Paramisgurnus dabryanus*	+	+	+	+	+	+	+	+	+	+	+	+
**Cyprinidae**												
18.*Rasbora steineri*					+	+	+	+	+			
19.*Zacco platypus*	+	+	+	+	+	+	+	+	+	+	+	+
20.*Opsariichthys bidens*	+	+	+	+	+	+	+	+	+	+	+	+
21.*Aphyocyprisarcus*				+	+	+	+	+				
22.*Mylopharyngodon piceus*								+			+	+
23.*Ctenopharyngodon idella*	+	+	+	+	+	+	+	+	+	+	+	+
24.*Elopichthys bambusa*									+	+	+	+
25.*Squaliobarbus curriculus*	+	+	+	+	+	+	+	+	+	+	+	+
26.*Chanodichthys erythropterus*	+						+	+		+	+	+
27.*Pseudolaubuca sinensis*	+			+				+	+	+	+	+
28.*Rasborinus lineatus*								+	+	+	+	+
29.*Parabramis pekinensis*								+	+	+	+	+
30.*Toxabramis houdemeri*			+	+	+	+				+	+	
31.*Hemiculter leucisculus*	+	+	+	+	+	+	+	+	+	+	+	+
32.*Hemiculterella wui*								+				
33.*Pseudohemiculter dispar*	+	+	+	+	+	+	+	+	+	+	+	+
34.*Pseudohemiculter hainanensis*				+	+	+	+	+	+			
35.*Megalobrama pellegrini*△												+
36.*Megalobrama terminalis*									+	+	+	+
37.*Culter alburnus*	+	+	+	+	+	+	+	+	+	+	+	+
38.*Culter recurviceps*	+	+	+	+	+	+	+	+	+	+	+	+
39.*Sinibrama macrops*			+	+	+	+	+	+	+	+	+	+
40.*Sinibrama melrosei*						+	+	+	+			
41.*Ancherythroculter lini*		+	+	+			+	+	+	+	+	+
42.*Distoechodon tumirostris*		+		+	+	+	+			+	+	+
43.*Xenocypris argentea*										+		
44.*Xenocypris davidi*	+	+	+	+	+	+	+	+	+	+	+	+
45.*Aristichthys nobilis*	+	+	+	+	+	+	+	+	+	+	+	+

Species	Sampling points											
	S1	S2	S3	S4	S5	S6	S7	S8	S9	S10	S11	S12
46.*Hypophthalmichthys molitrix*	+	+	+	+	+	+	+	+	+	+	+	+
47.*Hemibarbus medius*	+	+	+	+	+	+	+	+	+	+	+	
48.*Hemibarbus maculatus*		+	+	+	+	+	+	+	+			
49.*Hemibarbus labeo*	+	+	+									
50.*Pseudorasbora parva*	+	+	+	+	+	+	+	+	+	+	+	+
51.*Squalidus argentatus*		+	+	+	+	+	+	+	+	+	+	
52.*Abbottina rivularis*	+	+	+	+	+	+	+	+	+	+	+	+
53.*Microphysogobio elongata*								+				
54.*Saurogobio dabryi*	+	+	+	+	+	+	+	+				
55.*Gobiobotia kolleri*		+										
56.*Acheilognathus tonkinensis*	+	+	+	+	+	+	+	+	+	+	+	+
57.*Acheilognathus macropterus*				+	+	+	+	+				
58.*Rhodeus ocellatus*	+	+	+	+	+	+	+	+	+	+	+	+
59.*Barbodes semifasciolatus*			+	+	+	+	+	+				
60.*Spinibarbus denticulatus*				+	+	+	+	+	+	+	+	+
61.*Luciocyprinus langsoni*										+		
62.*Acrossocheilus parallens*						+	+	+	+			
63.*Acrossocheilus iridescens longipinnis*	+	+	+	+	+	+	+	+				
64.*Acrossocheilus hemispinus*				+	+	+	+					
65.*Acrossocheilus iridescens*				+	+	+	+					
66.*Onychostoma gerlachi*	+	+	+	+	+	+	+	+				
67.*Tor brevifilis*			+									
68.*Labeo rohita*△		+	+	+	+	+	+	+	+	+	+	+
69.*Cirrhinus molitorella*	+	+	+	+	+	+	+	+	+	+	+	+
70.*Cirrhinus mrigala*△		+	+	+	+	+	+	+	+	+	+	+
71.*Osteochilus salsburyi*	+	+	+	+	+	+	+	+	+	+	+	+
72.*Rectoris posehensis*			+	+	+	+	+	+				
73.*Semilabeo obscurus*					+							
74.*Ptychidio jordani*		+	+	+	+	+	+	+	+			
75.*Garra orientalis*	+	+	+	+				+	+	+	+	
76.*Discogobio yunnanensis*					+			+				
77.*Discogobio multilineatus*								+				
78.*Discogobio tetrabarbatus*	+	+	+	+	+	+	+	+				
79.*Pseudocrossocheilus bamaensis*	+	+	+	+	+	+						
80.*Cyprinus multitaeniata*			+	+	+	+	+	+	+			
81.*Cyprinus carpio*	+	+	+	+	+	+	+	+	+	+	+	+
82.*Carassius auratus*	+	+	+	+	+	+	+	+	+	+	+	+
**Balitoridae**												
83.*Liniparhomaloptera disparis qiongzhonqensis*					+					+		
84.*Paraprotomyzon bamaensis*					+							
85.*Beaufortia kweichowensis*								+				
86.*Sinogastromyzon wui*							+	+	+			
87.*Sinohomaloptera kwangsiensi*					+							
**Siluriformes**												
**Siluridae**												
88.*Pterocryptis anomala*	+	+	+	+	+	+	+	+	+	+	+	+
89.*Silurus asotus*	+	+	+	+	+	+	+	+	+	+	+	+
**Sisoridae**												
90.*Glyptothorax sinense*					+					+		
**Bagridae**												
91.*Tachysurus fulvidraco*	+	+	+	+	+	+	+	+	+	+	+	+
92.*Pelteobagrus intermedius*								+	+			
93.*Pseudobagrus vachellii*	+	+	+	+	+	+	+	+	+	+	+	+
94.*Pseudobagrus crassilabris*	+	+	+	+	+	+	+	+	+	+	+	+
95.*Hemibagrus guttatus*	+	+	+	+	+	+	+	+	+	+	+	+
96.*Hemibagrus macropterus*								+	+	+		
97.*Leiocassis longirostris*△	+	+					+	+	+	+	+	+
**Amblycipitida**												
98.*Liobagrus xiuenensis*					+							

Species	Sampling points											
	S1	S2	S3	S4	S5	S6	S7	S8	S9	S10	S11	S12
**Cranoglanididae**												
99.*Cranoglanis bouderius*									+	+		
**Clariidae**												
100.*Clarias fuscus*	+	+	+	+	+	+	+	+	+	+	+	+
101.*Clarias lazera*△	+	+	+	+	+	+	+	+	+	+	+	+
**Ariidae**												
102.*Arius sinensis*												+
**Loricariidae**												
103.*Hypostomus plecostomus*△								+	+	+	+	+
**Ictaluridae**												
104.*Ictalurus punctatus*△	+	+	+	+	+	+	+	+	+	+	+	+
**Mugiliformes**												
**Mugilidae**												
105.*Mugil cephalus*											+	+
**Beloniformes**												
**Hemirhamphidae**												
106.*Hyporhamphus intermedius*									+	+	+	
**Cyprinodontiforme**												
**Oryziatidae**												
107.*Oryzias latipes*								+	+			
**Poeciliidae**												
108.*Gambusia affinis*△							+	+	+	+	+	+
**Perciformes**												
**Serranidae**												
109.*Lateolabrax japonicus*												+
110.*Siniperca scherzeri*	+	+	+	+	+	+	+	+	+	+	+	
111.*Siniperca chuatsi*△	+	+	+	+	+	+	+	+	+	+	+	+
112.*Siniperca kneri*	+	+	+	+	+	+	+	+	+	+	+	+
113.*Coreoperca whiteheadi*					+	+			+		+	
**Centrarchidae**												
114.*Micropterus salmoides*△								+	+	+	+	+
**Cichlidae**												
115.*Oreochromis mossambicus*△			+	+	+	+	+	+	+	+	+	+
116.*Oreochromis niloticus*△	+	+	+	+	+	+	+	+	+	+	+	+
117.*Tilapia galilaea*△	+	+	+	+	+	+	+	+	+	+	+	+
118.*Coptodon zillii*△	+	+	+	+	+	+	+	+	+	+	+	+
119.*Parachromis managuensis*△			+	+	+							
**Odontobutidae**												
120.*Odontobutis sinensis*								+				
**Eleotridae**												
121.*Eleotris oxycephala*												+
122.*Sineleotris chalmersi*			+	+	+	+	+	+	+	+	+	+
123.*Neodontobutis hainanensis*								+				
**Gobiidae**												
124.*Glossogobius giuris*										+	+	+
125.*Rhinogobius giurinus*	+	+	+	+	+	+	+	+	+	+	+	+
126.*Rhinogobius duospilus*								+				
127.*Rhinogobius leavelli*	+				+			+				
128.*Rhinogobius filamentosus*				+	+							
**Anabantidae**												
129.*Anabas testudineus*					+	+	+	+	+	+		
**Belontiidae**												
130.*Macropodus ocellatus*								+				
131.*Macropodus opercularis*								+		+		
**Channidae**												
132.*Channa maculata*	+	+	+	+	+	+	+	+	+	+	+	+

Species	Sampling points											
	**S1**	**S2**	**S3**	**S4**	**S5**	**S6**	**S7**	**S8**	**S9**	**S10**	**S11**	**S12**
133.*Channa argus*	+	+	+									
134.*Channa gachua*			+	+	+					+	+	
135.*Channa asiatica*			+	+	+							
**Mastacembelidae**												
136.*Mastacembelus armatus*			+	+	+	+	+	+	+	+	+	+
**Synbranchiformes**												
**Synbranchidae**												
137.*Monopterus albus*	+	+	+	+	+	+	+	+	+	+	+	+
**Pleuronectiformes**												
**Cynoglossidae**												
138.*Cynoglossus trigrammus*											+	+
**Tetraodontiformes**												
**Tetraodontidae**												
139.*Takifugu ocellatus*									+	+	+	+

Note: △, Exotic species; +, Presence of the species recorded.
